# To determine the frequency of Factor V Leiden in cases of Deep Vein Thrombosis and Healthy controls

**DOI:** 10.12669/pjms.315.8088

**Published:** 2015

**Authors:** Anjum Saeed, Sumreen, Muhammad Ali Kashif

**Affiliations:** 1 Dr. Anjum Saeed, M.Phil Haematology, Frontier Medical College Abbotabad, Pakistan. Abbotabad - Pakistan; 2 Dr. Sumreen, M.Phil Haematology, SMBBMC Lyari Karachi, Karachi - Pakistan; 3 Dr. Muhammad Ali Kashif, FCPS, CMH Bahawalpur, Pakistan

**Keywords:** Factor V Leiden, Venous thromboembolism, Deep vein thrombosis

## Abstract

**Objective:**

To determine the frequency of Factor V Leiden in cases of Deep Vein Thrombosis and Healthy controls.

**Methods:**

This case control study was performed in Armed Forces Institute of Pathology Rawalpindi, From 21^st^ March to 25^th^ September 2013. One hundred patients with diagnostic evidence of Deep vein thrombosis on Doppler ultrasound/Magnetic resonance imaging (MRI) scan were included in the study through non probability convenient sampling and compared with 100 matched healthy controls. DNA was extracted from the blood sample by kit method. In order to identify Factor V Leiden mutation, the polymerase chain reaction (PCR) method was utilized combined with the Amplification refractory mutation system. Data was analyzed using statistical package for social sciences (SPSS) version 17.

**Results:**

In 100 patients of Deep Vein Thrombosis (DVT), frequency of Factor V Leiden (FVL) was 13% and it is was 2% in healthy control group. A significant association was found between FVL and DVT with odds ratio of 7.32 and with P value (P = 0.003).

**Conclusion:**

FVL was found to be highly prevalent among patients of DVT, Signifying strong association between the two.

## INTRODUCTION

Venous thromboembolism (VTE) is a disorder in which abnormal clotting of blood occur in deep veins of the body.^1^ Deep vein thrombosis and pulmonary embolism (PE) are the two main manifestations of venous thromboembolism.^1^ Symptomatic pulmonary embolism is present in about one third of patients with venous thrombosis.^1^ Venous thrombosis is widely considered as a disease of the west and therefore routine administration of thromboprophylaxis is not provided in Pakistan, even in those falling into the high-risk category.^2^ Exact data in terms of incidence or prevalence rate may be under reported owing to a number of factors, such as improper diagnosis, lack of diagnostic facilities (MRI/Doppler ultra sound) and lack of follow-up data in Pakistan.^2^

Deep veins of lower limbs are the most common site for generation of thrombi and dislodgment of these thrombi from their original position causes formation of emboli.^3^ Vessels of the pulmonary bed are a more common site for entrapment of these emboli as compared to brain, intestine and kidney. Therefore these sites for thrombosis are less common but the possibility is there.^3^

Factor V is a unique clotting factor that interacts in coagulation and anticoagulation at the same time.^4^ In its active form factor V acts as a cofactor stimulating factor Xa for the conversion of prothrombin into thrombin. Thrombin is then able to cleave the fibrinogen to fibrin and fibrin clot is formed. Once enough thrombin is produced, then to limit the process of clotting, Activated Protein C (APC) degrades the factor V and reduces further thrombin formation. Point mutation in Factor V generates a new variant called Factor V Leiden.^4^

Factor V Leiden has autosomal dominant inheritance. FVL polymorphism is specific for a point mutation at position 506 in which arginine is replaced by the glutamine. By this point mutation, sequence of amino acid where Activated Protein C acts is destroyed and renders the factor V resistant to degradation. Slow degradation causes longer coagulation process that is abnormal and leads to a hypercoagulable state.^4^

Venous thrombosis is a complex interplay of environmental, genetic and acquired factors, but it is idiopathic as well. Résistance to activated protein C due to Factor V Leiden and deficiencies of Protein C, S and Antithrombin III are the most common hereditary factors for venous thrombosis. Factor V Leiden has been found most frequent hereditary cause of venous thrombosis in whites of North America and in Europe. Moreover FVL is the most studied clotting factor worldwide as a cause of venous thrombosis.^5^

Management of venous thrombosis requires a thorough knowledge of diagnostic and treatment modalities. However an understanding of the underlying pathology and associated risk factors are equally essential.

The aim of this study was to compare the frequency of factor V Leiden between patients of Deep Vein Thrombosis and healthy population. It is necessary to determine the disease burden due to this mutation and provide better management for patients of recurrent thrombosis. Scarcity of data for factor V Leiden in Pakistan emphasized the need of more research in this area.

## METHODS

This case control study was conducted at the department of haematology Armed Forces Institute of Pathology Rawalpindi. From 21^st^ March to 25^th^ September 2013. Approval from ethical committee was obtained. Written consent was taken from all participants. Total 200 subjects were included in the study, 100 cases and 100 controls. Sampling was non-probability convenient. Cases were Patients of Deep vein thrombosis with diagnostic evidence of venous thrombo-embolism on Doppler ultrasound or MRI. Controls were healthy with no venous thrmbo-embolism. After selection detailed clinical history and demographic details were entered on a questionnaire. Number of thrombotic attacks, family history, and age were considered as potential risk factors. Patient age bracket was between 16 to 60 years.

Ethnic origin was only Punjabi from paternal and maternal sides. Equal number of age, sex and ethnicity matched healthy individual were selected as controls. Three ml venous blood was collected from each participant into Ethylene-diamine-tetra-acetic acid tube. DNA was extracted from the blood sample by kit method (cat. No. D 5001 Gentra USA). In order to identify Factor V Leiden mutation, the polymerase chain reaction (PCR) method was utilized combined with the Amplification refractory mutation system (ARMS).

Data was collected in the form of quantitative and qualitative variables and analyzed using statistical package for social sciences (SPSS) version 17. Mean and standard deviations were calculated for quantitative variables like age and number of thrombotic attacks. Frequency and percentages were calculated for qualitative variables like gender, family history of venous thromboembolism, and factor V Leiden. To determine the difference between the two groups in quantitative variables independent samples t-test was applied while for qualitative variables chi-square test was applied. P-value < 0.05 was considered as significant.

## RESULTS

Comparison of data between case and controls is shown in Table-I.

**Table-I:** Comparison of data between cases and controls.

	Cases	Controls	P-value
Age Mean (±SD)	42.53/(±8.2)	49.90(±9.69)	0.700
Gender	76 males	84 males	0.157
	24 Females	16 females	
*Number of thrombotic attacks*			
1	60 (60%)	0 (0%)	< 0.001
2	27 (27%)	0 (0%)	< 0.001
3	8 (8%)	0 (0%)	0.004
4	5 (5%)	0 (0%)	0.155
Family history for venous thromboembolism	4%	0%	0.043

### Factor V Leiden

In 100 cases FVL was present in 13 (13%) cases and absent in 87 (87%). While in controls FVL was present in only 2(2%) and absent in 98 (98%). Frequency of FVL was significantly higher in cases as compared to controls (p = 0.003, Odds ratio = 7.32).

Odds ratio was 7.32 indicating that those who have FVL are 7.32 times more at risk of developing venous thrombosis as compared to controls.

- P-value is 0.003 which shows that this association between FVL and venous thrombosis is statistically significant.
- 95% confidence interval of odd ratio was 1.15 - 48.40. This also proves that relationship was statistically significant.
- Moreover FVL is detected in only one patient of DVT with accompanying pulmonary embolism. Among 100 cases, 92 (92%) had only DVT while 8 (8%) had both DVT and pulmonary embolism.
- In FVL positive cases mean age was 39.5 years±10.6 and mean age of controls was calculated 29.5±7.07. P value is calculated 0.187.
- In FVL positive cases 09 are males and 04 are females. While in controls 02 males are FVL positive. P value is calculated 0.360.
- In 13 FVL positive cases only one has been found with positive family history. All the controls have negative family history. P value is calculated 0.687.
- In FVL positive cases and controls, numbers of thrombotic attacks have significant P value 0.002.

## DISCUSSION

We determined the frequency of FVL among patients of deep vein thrombosis and compared it with matched healthy controls to know the odds of FVL in DVT patients. The main aim of this study was to document the prevalence of FVL in Pakistan.

Prevalence of FVL varies from 0% to 15% according to ethnicity and geographic distribution worldwide.^6^ Prevalence of FVL mutation is higher in Caucasian than non Caucasian and low in African, Asian and South European populations (1%–3%). It is relatively high in North America (5%) and very high in Mediterranean populations (13.6% in Syria, 12.3 % in Jordan, and 13.4% in Greece).^7^ Venous thromboembolism due to FVL is usually considered the disease of West especially in Europe and it is considered almost nil in Asians but recent research showing different results. A study in Iran by Irani-Hakime claimed almost the same frequency of FVL in East as in the West.^8^ A similar study in North India by Garewal showed 12% FVL in DVT patients.^9^ Study in Iran by Rahimi showed the FVL present 11.4% cases of DVT.^10^

In our study, FVL mutation was found to be 13% among the cases of VTE while it is 2% in healthy controls. The mutant allele is highly expressed among Pakistani patients with DVT. There are two local studies in which FVL has been studied. In a study by Nassirudin the frequency of FVL was 1.3%,^11^ although this 1.3% prevalence does not match with our study but reason may be the difference in target population. Moreover 2% FVL was detected in healthy controls which showed high prevalence of FVL. Our finding of high frequency of FVL in VTE patients is supported by another study that conducted in Aga Khan Hospital Karachi by Khalid which revealed FVL present 14.1% cases of venous thrombosis.^12^ Our study emphasizes that more research is required on larger scale and it is important to perform test for FVL mutation in cases of DVT.

Mean age of our cases and controls is similar to other studies. Several studies have claimed the incidence of VTE due to FVL over the age of 40 years.^13^ Our finding suggests that men are more prone to develop VTE as compared to women. This finding of gender association in our study is agreement with previous research.

Our study also showed recurrent DVT in the Carrier of FVL. It is evident through various studies that risk of recurrent thrombotic attacks is significantly higher in carriers of FVL than in patients without this abnormality.^14^ Ridker emphasizes the need of more prolong anticoagulation in presence of FVL (heterozygous).^14^ Unfortunately no local study is available for comparison, but international data supports our finding that FVL increases the risk of recurrent DVT.^14^

Positive family history is important, it can be considered as independent risk factor for DVT, regardless of other pro-thrombotic factors. Our study showed that family history of FVL is not significant. International data does not support our finding and this discrepancy should be evaluated with a larger study in order to overcome the limitations of our study.

In our study, 8% patients had DVT with PE while 92% patients had DVT alone. This shows that PE is less frequent complication of FVL. FVL paradox in DVT and PE has been studied in several studies and is still under research.^15^ There are mixed results about this paradox, some are similar to our study and few are different.

Thrombotic events in patients with heterozygous FVL mutation are approximately 10%.^16^ In our study all the positive cases of FVL were heterozygous. No case of homozygous allele was detected.

On the basis of our results, we suggest that patients of venous thrombosis should be screened for FVL detection as a cause of thrombosis. In the absence of triggering factors, thrombosis predominantly of lower limb should be evaluated for FVL detection either by screening or by PCR.

Our study denies the usual concept of low prevalence of FVL in Asians. Regarding the high frequency in DVT patients, family members of any subject with FVL mutation should be screened. Further investigations are needed to explore the other areas of interest regarding FVL in thrombogenesis.

### Limitations of the study

Most of the patients were illiterate therefore data about family history was difficult to extract. Same was true for recurrent thrombotic attacks. Therefore discrepancies regarding family history and recurrent thrombotic attacks are present but genetic and geographic alterations may also be responsible.

## CONCLUSION

FVL was found to be highly prevalent among patients of DVT, Signifying strong association between the two. Thus FVL must be screened in all patients with venous thromboembolism.
